# A Word Pair Dataset for Semantic Similarity and Relatedness in Korean Medical Vocabulary: Reference Development and Validation

**DOI:** 10.2196/29667

**Published:** 2021-06-24

**Authors:** Yunjin Yum, Jeong Moon Lee, Moon Joung Jang, Yoojoong Kim, Jong-Ho Kim, Seongtae Kim, Unsub Shin, Sanghoun Song, Hyung Joon Joo

**Affiliations:** 1 Department of Biostatistics Korea University College of Medicine Seoul Republic of Korea; 2 Korea University Research Institute for Medical Bigdata Science Korea University Seoul Republic of Korea; 3 Department of Cardiology Cardiovascular Center Korea University College of Medicine Seoul Republic of Korea; 4 Department of Linguistics Korea University Seoul Republic of Korea; 5 Korea University Research Institute for Medical Bigdata Science Korea University Anam Hospital Seoul Republic of Korea; 6 Department of Medical Informatics Korea University College of Medicine Seoul Republic of Korea

**Keywords:** medical word pair, similarity, relatedness, word embedding, fastText, Korean

## Abstract

**Background:**

The fact that medical terms require special expertise and are becoming increasingly complex makes it difficult to employ natural language processing techniques in medical informatics. Several human-validated reference standards for medical terms have been developed to evaluate word embedding models using the semantic similarity and relatedness of medical word pairs. However, there are very few reference standards in non-English languages. In addition, because the existing reference standards were developed a long time ago, there is a need to develop an updated standard to represent recent findings in medical sciences.

**Objective:**

We propose a new Korean word pair reference set to verify embedding models.

**Methods:**

From January 2010 to December 2020, 518 medical textbooks, 72,844 health information news, and 15,698 medical research articles were collected, and the top 10,000 medical terms were selected to develop medical word pairs. Attending physicians (n=16) participated in the verification of the developed set with 607 word pairs.

**Results:**

The proportion of word pairs answered by all participants was 90.8% (551/607) for the similarity task and 86.5% (525/605) for the relatedness task. The similarity and relatedness of the word pair showed a high correlation (*ρ*=0.70, *P*<.001). The intraclass correlation coefficients to assess the interrater agreements of the word pair sets were 0.47 on the similarity task and 0.53 on the relatedness task. The final reference standard was 604 word pairs for the similarity task and 599 word pairs for relatedness, excluding word pairs with answers corresponding to outliers and word pairs that were answered by less than 50% of all the respondents. When FastText models were applied to the final reference standard word pair sets, the embedding models learning medical documents had a higher correlation between the calculated cosine similarity scores compared to human-judged similarity and relatedness scores (namu, *ρ*=0.12 vs with medical text for the similarity task, *ρ*=0.47; namu, *ρ*=0.02 vs with medical text for the relatedness task, *ρ*=0.30).

**Conclusions:**

Korean medical word pair reference standard sets for semantic similarity and relatedness were developed based on medical documents from the past 10 years. It is expected that our word pair reference sets will be actively utilized in the development of medical and multilingual natural language processing technology in the future.

## Introduction

The rapid development of natural language processing (NLP) technology in tandem with advances in artificial intelligence and deep learning have greatly influenced our day-to-day life. In the field of medical service, there are high expectations that NLP will be able to improve patient-physician communication and provide basic medical information support for patients in the blind spot of medical care. Several commercial chatbot applications are now available on the web or mobile environments [1]. For example, OneRemission provides useful information for patients with cancer. Babylon Health also provides symptom-based medical consultation services.

However, medical terms require special expertise and are challenging to decipher not only for ordinary people but also, at times, for medical experts. This raises 2 fundamental requirements with regard to medical NLP technology. First, as medical terms appear sparsely in plain text, a vast amount of medical-specific text data is required to improve the current medical NLP technology. Second, as medical terms often convey a wide range of meanings in different contexts, it is crucial to build a semantic network based on a comprehensive set of medical terminologies via word embedding. For example, BioWordVec is a set of biomedical word embeddings involving 2,324,849 words from Medical Subject Headings (MeSH) and PubMed [2]. BioConceptVec was created by learning domain-specific vector representations of biological concepts (eg, genes, drugs, and proteins) using large-scale biomedical corpora [3]. BioBERT, a biomedical-specific language model, was constructed using approximately 18 billion words from PubMed abstracts and PubMed Central full-text articles [4]. Although English is the main language being used in the field of medical NLP, multilingual approaches involving other languages (eg, Chinese, German, French, Italian, Japanese, Korean) are also being investigated [5,6]. Technical validation of language embedding models is also highly important in the field of medical NLP. Only a few standard datasets have so far been introduced. University of Minnesota Semantic Relatedness Set (UMNSRS) datasets (566 pairs for similarity and 587 pairs for relatedness) were developed by involving 8 medical residents [7]. Previously, a Mayo semantic relatedness set of 101 medical term pairs evaluated by 13 medical coding experts was proposed [8]. Both of the aforementioned datasets were created more than a decade ago. Many important medical discoveries have been made over the past decade, and as a result, the medical procedures have also undergone changes. Therefore, the erstwhile reference standards do not necessarily involve the current knowledge of medical science. This raises the necessity of updating the standard datasets that are being used for NLP model validation.

This study aimed to propose a new standard word pair set especially for Korean medical terms. We generated a large amount of text data using Korean medical terms through academic papers, websites, and textbooks and selected words in consideration of the frequency of appearance of each word. Concept ratings for the new standard word pair set were set by highly qualified attending physicians from a tertiary hospital. Finally, we evaluated the developed word embedding model to demonstrate its feasibility.

## Methods

### Data Acquisition

We collected 3 types of Korean medical documents. Medical research articles were selected as high-quality documents at the professional level, health information news articles were selected as popular and general documents, and medical textbooks were selected as intermediate level but very high-quality documents. First, regarding medical textbooks, 2 Korean publishing companies provided textbooks for the present study. Each publisher had a classification system for the subject of books and textbooks in the 54 subfields (eg, internal medicine, emergency medicine, orthopedics, dentistry, pharmacy, public health) of the medical field that were selected. Finally, 518 text files from Korean medical textbooks published from 2010 to 2020 were used. Second, regarding health information news, NAVER, a widely used internet portal site in Korea, distributes news articles from general newspapers, internet newspapers, and broadcasting stations. We collected all the news articles in a section called “Health Information” under the “News” section of the NAVER portal. Finally, 72,844 health information news articles published from January 1, 2010 to December 31, 2020 in NAVER were collected through internet crawling. Third, regarding medical research articles, 72 journals (eg, Journal of The Korean Society of Integrative Medicine, Journal of The Korean Society of Emergency Medicine) published in the Korean language were selected from the journals listed in the Korean Studies Information Service System (KISS). Finally, 15,698 medical research articles published from 2010 to 2020 were collected. The text files were parsed and modified for further investigation. Consequently, we were able to build a large corpus comprising 191 million tokens in terms of morphemes (129 million tokens in terms of Korean words).

### Pair Set Development

The top 10,000 nouns were selected in order of occurrence frequency in the corpus. Those terms were then categorized by 2 experienced medical vocabulary experts (a certified health information manager and a medical physician) into the following 5 categories: “symptom and sign,” “diagnosis,” “medication,” “operation and procedure,” and “not applicable.” Because the 10,000 nouns included nonmedical terms, the “not applicable” items were manually filtered out. This process left 1214 medical terms in total (625 diagnoses, 277 symptoms and signs, 177 medications, 135 operations and procedures). After the medical term selection, 607 medical term pair sets were manually developed considering the distribution of similarity and relatedness for each pair set. The similarity and relatedness for each pair set were categorized into 4 groups (very dissimilar/unrelated, somewhat dissimilar/unrelated, somewhat similar/related, and very similar/related). The order of presentation of the pair sets and the order of the terms in each pair set were randomized for each participant.

### Human Validation

For the validation of the medical term pair set, 16 attending physicians (2 cardiologists, 1 gastroenterologist, 3 nephrologists, 1 endocrinologist, 1 oncologist, 1 infectious medicine doctor, 1 family medicine doctor, 2 pediatricians, 1 psychiatrist, 1 emergency medicine doctor, 1 radiologist, and 1 general surgeon) at Korea University Anam Hospital were recruited. The study protocol was approved by the Institutional Review Board of Korea University Anam Hospital (IRB No. 2021AN0059). Written informed consent was obtained from all participants at enrollment. Our study complied with the principles of the Declaration of Helsinki.

The participants were randomized into 1 of 2 tasks (similarity or relatedness); 8 participants were assigned to the similarity evaluation group, and the other 8 were assigned to the relatedness evaluation group. The tasks were explained to the participants with examples (eg, “myocardial infarction” and “chest pain” are related but not similar; “cardiovascular disease” and “coronary artery disease” are similar). The 8 participants were separated into quiet rooms, where they sat in front of a 15-inch laptop. Relatedness and similarity evaluations for each medical term pair set were performed on a laptop using a toolkit used in psychological experiments known as OpenSesame [9]. The evaluations were performed using a 10-point scale, ranging from 1 to 10: the greater the value, the higher the similarity or relatedness [10]. In the case of a word pair set that was difficult to answer, the participants were asked to skip it by entering “x.” In the case of word pair sets from different semantic domains, there was a possibility that the participants may unknowingly answer the relatedness rather than the similarity, especially owing to time constraints. This could lead to a somewhat low degree of correspondence in terms of scoring in the similarity task. Therefore, to minimize the potential error and bias, no time limit was given for answering. However, the subjects were instructed to answer the word pairs in an intuitive manner. To maintain the degree of concentration on the task, after each 200 word pairs, the participant was allowed to autonomously take a break of 3-5 minutes. To minimize practice effects, a practice session consisting of 15-24 word pairs not included in the main word pair sets was provided before the main evaluation (Table 1).

**Table 1:** Examples of the practice session for human validation in which word pairs of the practice session included medical as well as general terms. Term 1 and Term 2 were presented to the participants. However, their anticipated similarity and relatedness categories were kept hidden.

Term 1	Term 2	Anticipated category
책방 (bookstore)	서점 (bookshop)	Similarity: high
학교 (school)	경찰서 (police station)	Similarity: middle
까치 (magpie)	중국어 (the Chinese language)	Similarity: low
친구 (friend)	사람 (human)	Relatedness: high
겨울 (winter)	난로 (heater)	Relatedness: middle
핸드폰 (cell phone)	미술 (art)	Relatedness: low
심혈관질환 (cardiovascular disease)	관상동맥질환 (coronary artery disease)	Similarity: high
암성통증 (cancer pain)	월경통 (menstrual pain)	Similarity: middle
좌골신경통 (sciatica)	간성혼수 (hepatic coma)	Similarity: low
심근경색 (myocardial infarction)	흉통 (chest pain)	Relatedness: high
세티리진 (cetirizine)	구강건조 (dry mouth)	Relatedness: middle
백반증 (vitiligo)	라미 (lamisil)	Relatedness: low

### Embedding Model Validation

We applied 2 unsupervised word embedding models (Word2Vec [11] and FastText [12]) to the Korean medical word pair sets, and the results were compared with those of the human evaluation. A Korean medicine–focused corpus with 129 million words (aforementioned) was used for model training. The preprocessing of the obtained corpus was twofold. First, the entire corpus data were segmented using the Korean Sentence Splitter (KSS) 2.2.0.2. Second, the Mecab-ko tagger 0.4.0 was used to convert the sentences into morphological tokens [13]. Then, the models were built using the Gensim Python library [14]. The details related to the tuning of the model hyperparameters are listed in Table 2.

The cosine distance between the embedded concepts of word pairs was calculated and compared with that of the human evaluation.

**Table 2:** Hyperparameters of Word2Vec and FastText.

Parameter name	Specified argument
Dimension size	300
Window size	5
Negative sampling ratio	10%
Minimum frequency	10
Workers	3
Batch words	10,000
Alpha	0.25%
Epochs	20

### Statistical Analyses

**Figure 1:**
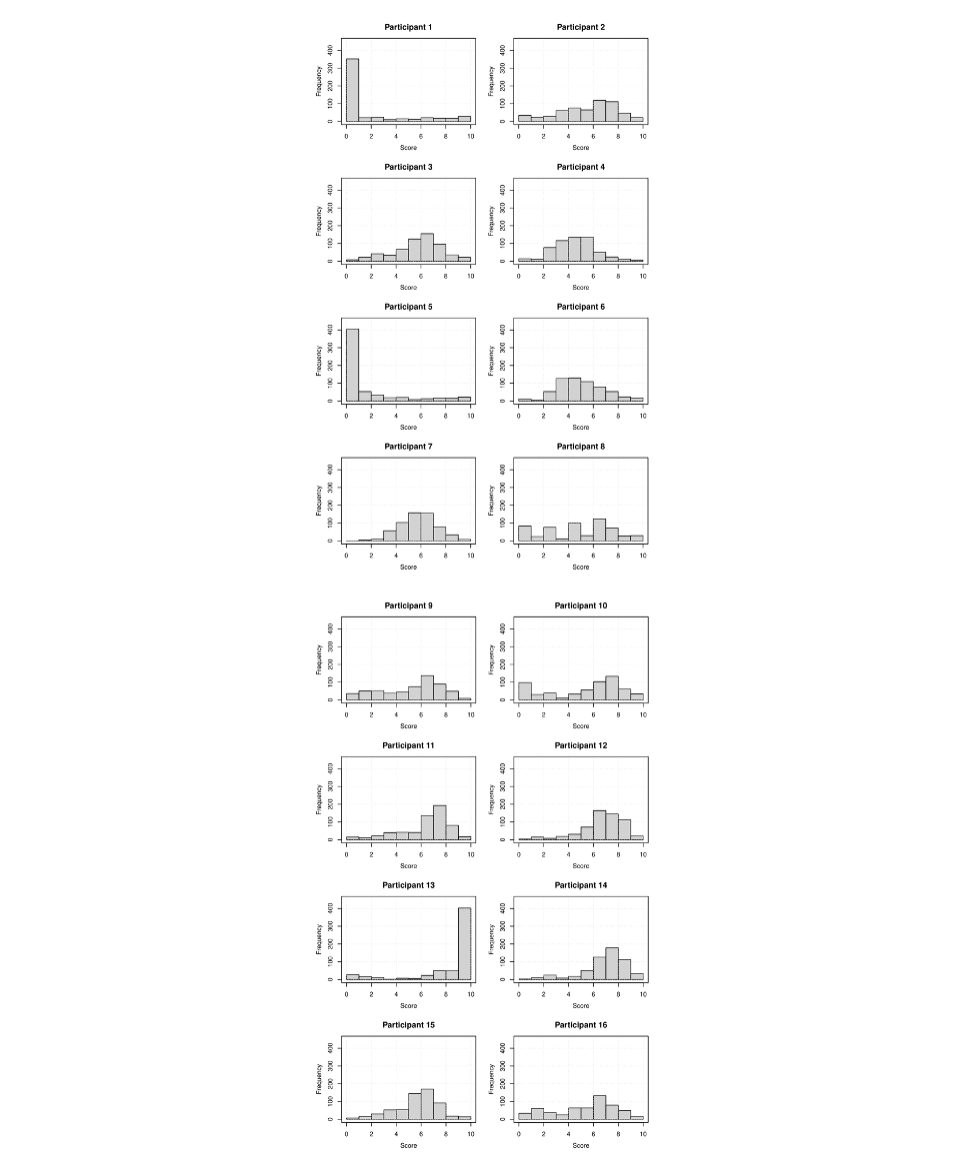
Score distribution plots of the participants (n=16 attending physicians from a tertiary hospital); the distributions of the scores of participants 1, 5, and 13 were absolutely skewed and were therefore excluded from further analyses.

The word pair set was organized in a variety of ways, ranging from nonsimilar/related to closely similar/related. Although the participants received instruction, the distribution of the scores of some participants was found to be highly skewed (Figure 1). The scores of the participants whose absolute skewness value of the score distribution was 1.5 or higher were excluded. The scores of 6 participants in the similarity task and 7 participants in the relatedness task were finally included for further analyses.

The intraclass correlation coefficient (ICC) was calculated to measure the interrater reliability. Among the models defined by Shrout and Fleiss [15], the ICC(2,1) was used. This model is a two-way random effects model based on a single rater used for generalizing reliability results. The definition of consistency was chosen to consider the scores of the subjects that were correlated in word pairs. To compare the distribution between the original and modified data, the Kolmogorov-Smirnov statistic was used. Spearman rank correlation was used to determine the relationship between similarity and relatedness and to compare the magnitude of the automated measures of relatedness and similarity representing the human annotated scores.

## Results

Words in the standard reference dataset should be sufficiently representative of medical terms, and if so, they should show a high response rate. The previous UMNSRS dataset showed 81.1% (587/724) and 78.2% (566/724) response rates on the relatedness and similarity tasks, respectively [7]. This study revealed that the word pair sets scored by all the assigned participants on the relatedness task were 86.5% (525/607) of the 607 word pair sets, and on the similarity task, these were 90.8% (551/607) of the 607 word pair sets. This indicates that the words in the present dataset have some degree of representation as medical terms. The most common scoring failure cases were word pair sets including “diagnosis” category words on both relatedness and similarity tasks (99/153, 64.7% and 51/77, 66% of all failures for relatedness and similarity tasks, respectively). This failure to score can be attributed to the fact that diagnostic approaches are rapidly developing and becoming increasingly specialized. The highest failure rate of scoring for the diagnosis-related word pairs in this study is believed to reflect the rapid pace of development in the medical field. Although some word pairs were not scored, all 607 word pair sets were scored by more than 4 (4/8, 50%) participants in each task group. The average response time per word pair set was 4.4 (SD 2.9) seconds on the relatedness task and 3.0 (SD 2.4) seconds on the similarity task. In the relatedness and similarity tasks, 71.8% (3050/4249) and 90.2% (3284/3642), respectively, of all the responses were completed within 5 seconds. This indicates that most of the responses were answered in an intuitive manner.

**Figure 2:**
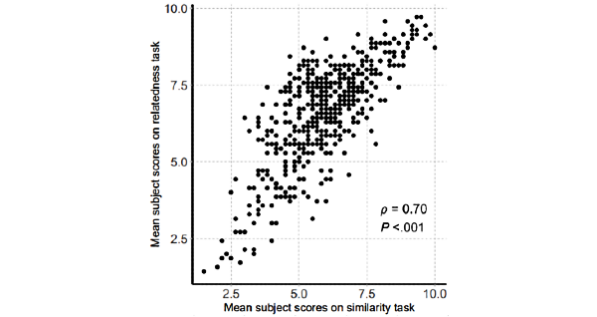
Scatter plot of the correlation between the similarity and relatedness tasks.

The scores between relatedness and similarity were highly correlated (*ρ*=0.70, *P*<.001; Figure 2). Mean score was higher in the relatedness task as compared to the similarity task (6.6, SD 1.6 vs 5.9, SD 1.5; *P*<.001). Further, compared to relatedness, it was difficult to assess the similarity between medical terms that were from different semantic domains (eg, “myocardial infarction” [disease domain] and “percutaneous coronary intervention” [procedure domain]). Therefore, several word pairs, which can be considered as somewhat similar or even highly related, were determined as dissimilar (upper left side of the plot in Figure 1).

To use the present word pair sets as a reference standard, the consistency of the scores must be evaluated. The interrater agreements on the word pair sets were in an acceptable range (ICC=0.53 and 0.47, in the relatedness and similarity tasks, respectively, *P*<.001). Previously, word pairs in the UMNSRS sets showed different patterns of disagreement based on their domains [7]. The word pairs from the same domain (Drug-Drug) showed higher ICC compared to the other domain categories in the UMNSRS sets. Similarly, the disagreements related to scoring on the word pair sets were not uniformly distributed in this study. In the similarity task, interrater agreement of the word pair sets consisting of the same domain was higher than that of the word pair sets consisting of the different domains. However, in the relatedness task, word pair sets consisting of the different domains had higher interrater agreement (Table 3). To qualify the reliability and consistency of the scores, the word pairs that were scored by more than half of the participants and the SDs of the scores higher than any values above 1.5 × interquartile ranges were removed from the original word pair sets. After removing 3 word pairs in the similarity task and 8 word pairs in the relatedness task, 604 word pairs in the similarity task and 599 word pairs in the relatedness task were included in the final standard reference Korean medical word pair set (Multimedia Appendix 1 and Multimedia Appendix 2). There was no difference in the interrater agreements between the word pair sets in the final word pair sets and the original word pair sets. The distribution of scores was also similar between the original and final word pair sets in both tasks (Kolmogorov-Smirnov statistic for both tasks, *P*>.90).

**Table 3:** Interrater agreement (using the intraclass correlation coefficient) on word pair-sets grouped by the semantic domain types.

Task		Word pairs of the same domain	Word pairs of different domains
**Similarity task**			
	Original word pair set	0.49^a^	0.41^b^
	Final word pair set after modification	0.49^c^	0.42^d^
**Relatedness task**			
	Original word pair set	0.52^a^	0.57^b^
	Final word pair set after modification	0.51^e^	0.57^f^

^a^n=409.

^b^n=198.

^c^n=408.

^d^n=196.

^e^n=407.

^f^n=192.

**Figure 3:**
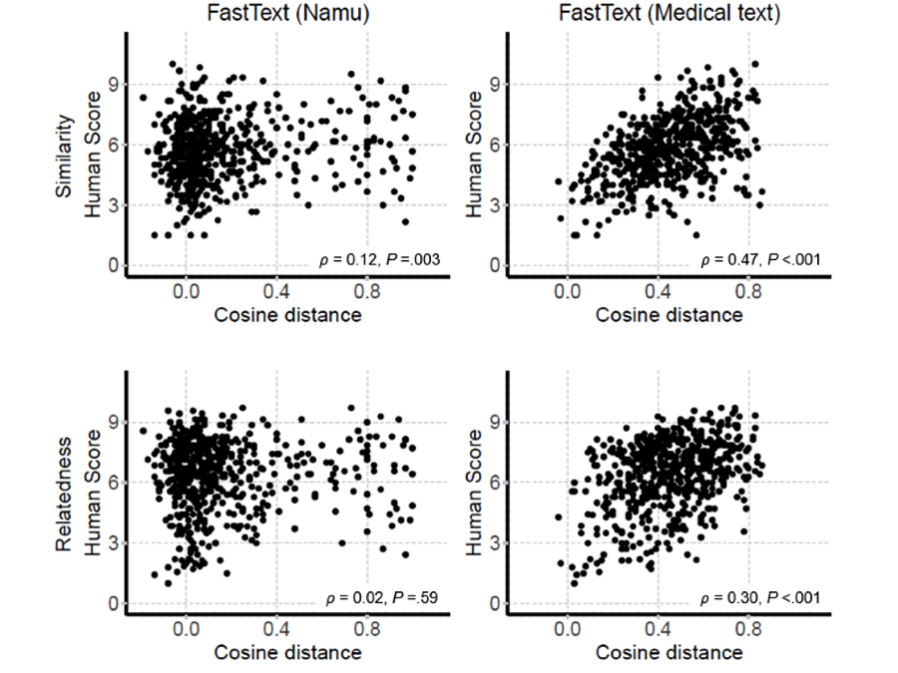
Correlation between cosine distance from FastText and the human evaluations from 13 attending physicians.

To explore the application and efficacy of the final word pair sets, they were applied to the 2 most used unsupervised word embedding models (Word2Vec and FastText). All the word pairs were successfully retrieved by the FastText trained with namu and medical text, and 13 (13/607, 2.1%) word pairs with namu and 145 (145/607, 23.9%) word pairs with medical text were successfully retrieved by Word2Vec. Because more than half of the word pairs were excluded from the Word2Vec model, we only focused on the FastText model. The correlations between cosine distance and human evaluation were higher in the models trained using medical text than in those trained using namu (Figure 3). These results suggest that collection and application of Korean medical texts are key to the development of NLP technology in the Korean medical field.

## Discussion

In this study, a standard Korean medical word pair set is proposed, with reference values for the similarity and relatedness of word pairs. The novel contributions of this study are as follows: (1) The proposed word pair set used contemporary medical words from various resources, such as textbooks, academic journals, news, and social media; (2) the similarity and relatedness were assessed by attending physicians with at least 5 years of experience in various fields; (3) the developed set is the Korean version and also the first non-English standard reference dataset for semantic similarity and relatedness; (4) when this set was tested for different embedding models, it was found that the more medical-specific the embedding models, the higher the similarity scores provided by the physicians. Therefore, the present word pair set can be successfully applied to evaluate how well the embedding models can represent the medical concepts.

### Comparison With Prior Work

Several reference standards exist for estimating semantic relatedness. MayoSRS and MiniMayoSRS consist of 101 and 29 clinical word pairs, respectively, whose relatedness was estimated by 9 medical coders and 3 physicians [8,10]. UMNSRS-Similarity and UMNSRS-related datasets, which were developed in 2010, consist of 566 and 587 word pairs of unified medical language system (UMLS) concepts [7], respectively. Their corresponding similarity and relatedness scores were manually assessed by 8 medical residents. Several studies have adopted these datasets [16,17]. The other reference standard involves the random selection of word pairs from the standardized Medical Dictionary for Regulatory Activities queries [18]. Semantic similarity and relatedness were automatically calculated based on the previously proposed statistical formulas (eg, Resnik [19] and Lin [20]) and the probability sources from the Adverse Event Reporting System database. UMLS and MedDRA are standardized medical vocabularies with organized code systems. However, they are limited because they do not reflect the terms used in the real world. The text sources of this study included textbooks, academic journals, news, and social media articles. Moreover, medical terms that appeared frequently were selected to better reflect the reality of actual use in the present era. In addition, the word pair set of this study include newer terms, such as “Middle East Respiratory Syndrome (MERS),” “Apixaban,” and “Keytruda.”

### Korean Translation Version of UMNSRS Word Pair Sets

Before a reference standard word pair set for a local language of Korean was proposed in this study, we considered using a word pair set of an English reference standard, such as the UMNSRS data set, translated into a Korean version. In a preliminary study with 12 health information managers certified by the Korean government (similar to the registered health information administrators or technicians in the United States), the average answer rate for the UMNSRS word pair sets translated into Korean was only 66.7% for the similarity set and 66.5% for the relatedness set. The average answer rates of the same participants for the present word pair sets were increased to 81.5% for the similarity set and 82.1% for the relatedness set. There could be 2 underlying reasons for the difference in response rates: the translation task and the medical environment.

First, the translation process inevitably causes some data loss, even across Indo-European languages. In a French study, the UMNSRS word pair sets were translated into French, and only 73% of the similarity set and 71% of the relatedness set were translated and used [21]. In a comparable Spanish study, only 65% of the relatedness set and 67% of the similarity set were automatically rendered into Spanish because of regional differences in medical protocols and commercial drug names [22]. These reports imply that reference standards need to be developed specifically for each language.

**Figure 4:**
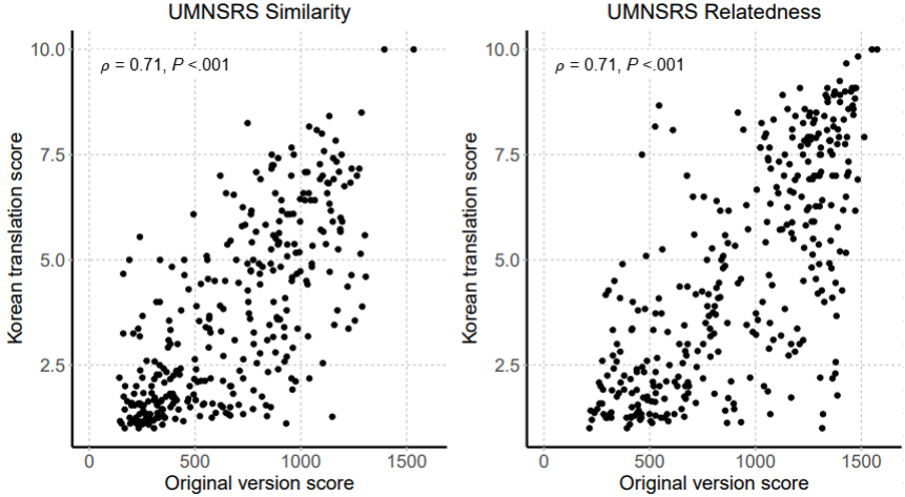
Correlation between the reference scores for the original University of Minnesota Semantic Relatedness Set (UMNSRS) word pair sets (English version) and the scores from 12 health information managers for the Korean translation version.

**Figure 5:**
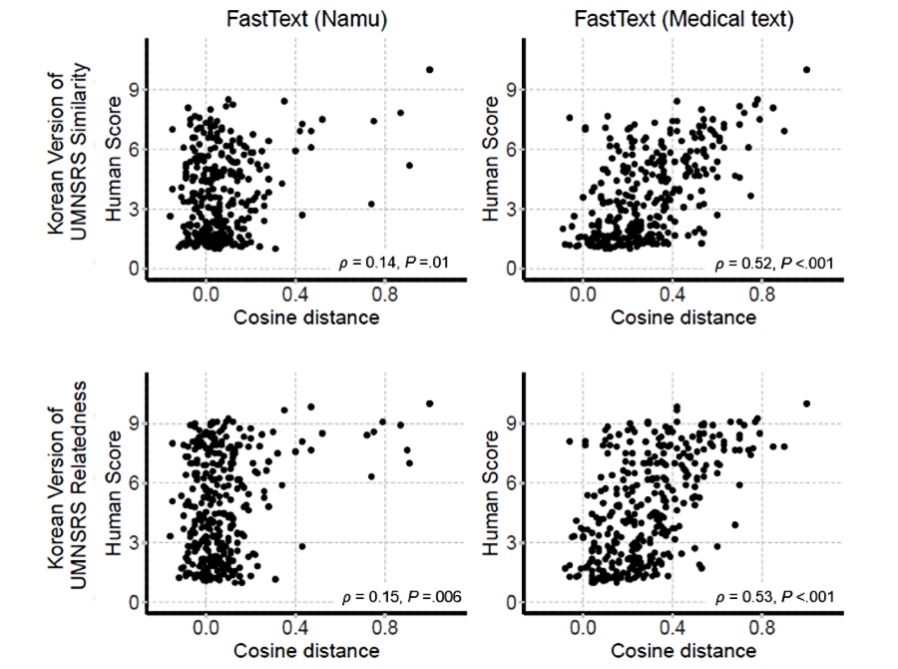
Correlation between the cosine distance of FastText embedding models and the human evaluations by 12 health information managers of the Korean version of the University of Minnesota Semantic Relatedness Set (UMNSRS) word pair sets.

Second, differences in cultural and medical backgrounds may influence the response rate. The correlations between the scores given for the Korean translation and for the original UMNSRS word-sets were modest (*ρ*=0.71 for the similarity and 0.71 for relatedness; Figure 4). When the embedding models in the present study were applied to the Korean translation version of UMNSRS word pair sets, the correlations between the cosine distance of the embedding models and the human evaluation scores of 12 health information managers on Korean-translated UMNSRS word pair sets were similar for the similarity task and slightly higher for the relatedness task compared to the correlations between the cosine distance of the embedding models and the scores of 13 attending physicians on the reference standard word pair sets from the present study (Figure 5). Notably, human evaluations were performed in different groups (health information managers and attending physicians). In word pair scoring, experienced physicians could be more affected by the complexity and various possibilities of medicine. This can be attributed to the relatively inconsistent results for the tasks, particularly the relatedness task. Furthermore, the semantic relation of word pairs in the present study could be more complex and specialized compared to those of UMNSRS word pair sets. This is bolstered by the fact that the correlation between the cosine distance of the embedding model trained with only namu wiki and the scores of physicians on the reference standard word pair sets of the present study was extremely low for the relatedness task (*ρ*=0.02). It is also noteworthy that the word embedding model trained with the medical texts shows better performance on both the word pair sets (Korean translated UMNSRS word pair sets and the word pair sets of the present study). The results thus obtained suggest that it is important to secure large medical texts for better performance in word embedding techniques.

Considered together, it is expected that the Korean word pair sets proposed in the present study can be used as one of the global reference standards through appropriate translation along with the UMNSRS word pair sets. We acknowledge that the UMNSRS word pair sets have the potential to become a global reference standard though appropriate translation.

### Multilingual NLP

Recently, a variety of NLP technologies for diverse human languages have been studied. However, there are only a few studies on multilingual NLP in the medical field [23-25]. Some studies built their own corpus and compared the performance of their embedding models [25,26]. One study adopted a metathesaurus (eg, UMLS) that included multiple languages [25]. Using a reference standard for each language to evaluate multilingual NLP models is not preferred because of the following 2 reasons. First, because the translation task itself is neither straightforward nor bias-free, we cannot necessarily rely on translated data. Second, to the best of our knowledge, no word pair dataset has yet been built for use as a non-English reference standard.

An ideal reference standard pertains to the underlying linguistic structures of an individual language, as well as the semantics of medical concepts. It has been noted that recent multilingual NLP architectures involve some elementary cross-linguistic knowledge [26,27]. However, linguistically naive NLP models often do not consider the typological differences in different languages (eg, morphological features) because they are overfitted to a specific type of language variation. Thus, linguistically naive multilingual models fail even in a word-by-word evaluation [28]. Furthermore, a multilingual NLP system is prone to biases because large English corpora are heavily weighted against low-resource languages, such as Korean corpora. This data scarcity problem undermines the reliability of word embeddings trained on medical texts and causes the model to perform inconsistently across different languages.

### Limitations

The present study has several limitations. First, the most noticeable discrepancy between human cognition and embedding models is based on semantics. For example, 이부프로펜 (ibuprofen) is one of the most popular nonsteroidal anti-inflammatory drugs. The participants in our study could easily recognize the word 발열 (fever) and 이부프로펜 (ibuprofen). However, this drug was not considered when building our corpus owing to the lack of sufficient reasons. Instead, the word 이부프로펜 (ibuprofen) was usually identified by its effects, such as 해열 (anti-fever) and 소염 (anti-inflammation). As a result, the cosine similarities between 이부프로펜 (ibuprofen)-발열 (fever) was 0.18, which is considerably lower than 이부프로펜 (ibuprofen)-해열 (anti-fever), at 0.53, and 이부프로펜 (ibuprofen)-소염 (anti-inflammation), at 0.61. This implies that word embedding models are not good at finding similarities between words with low distributional and high lexical analogy. Second, capturing intuitive judgments by medical experts was not directly correlated to the automatic prediction of medical labels, such as the names of treatments and drugs. Thus, the Korean version of similarity and relatedness data in this study regards that word embeddings represent human-like knowledge of Korean medical terms, whereas they are general predictors of performance in real-world applications. This distinction between intrinsic and extrinsic evaluation also corresponds to the standard methodology for assessing unsupervised word embeddings trained on general domain corpora [29]. Third, there was bias in the preliminary results from the Korean translation of UMNSRS word sets presented in the Discussion section. The medical specialty and domain knowledge of the participating group between the Korean translation and the original version were not met. Further, no experiment was conducted involving multilingual participants. Although these results are insufficient to reveal the limitations of translation, and since the current translation technology is rapidly developing, limitations related to translation in specialized fields, such as medical terms, still exist. In this respect, we believe that developing reference standards for various languages can help accelerate the development of medical NLP technologies in the future.

### Conclusions

The word pair reference standard is an important tool for evaluating the performance of NLP techniques, such as word embedding models. It is difficult to evaluate the semantic relevance of medical terms, as such evaluations require knowledge and expertise. In this study, 604 word pairs were proposed for similarity evaluation and 599 word pairs for relatedness evaluation as Korean reference standard word pair sets for use in the medical domain. This study is the first step toward the development of a word pair reference standard using various resources, including textbooks, news, and academic journals published in a non-English language, Korean, and is expected to facilitate the further acceleration of medical NLP techniques for different languages.

### Data Availability

The final word pair sets for the similarity and the relatedness are available at https://github.com/KU-RIAS/.

## Appendix

Multimedia Appendix 1 Medical word pair set (similarity, Korean).

Multimedia Appendix 2 Medical word pair set (relatedness, Korean).

## Abbreviations

ICC: intraclass correlation coefficient
KHIDI: Korea Health Industry Development Institute
KISS: Korean Studies Information Service System
KSS: Korean Sentence Splitter
MERS: Middle East Respiratory Syndrome
MeSH: Medical Subject Headings
NLP: natural language processing
UMLS: unified medical language system
UMNSRS: University of Minnesota Semantic Relatedness Set

## References

[1] Safi Z, Abd-Alrazaq A, Khalifa M, Househ M (2020). Technical Aspects of Developing Chatbots for Medical Applications: Scoping Review. J Med Internet Res.

[2] Zhang Y, Chen Q, Yang Z, Lin H, Lu Z (2019). BioWordVec, improving biomedical word embeddings with subword information and MeSH. Sci Data.

[3] Chen Q, Lee K, Yan S, Kim S, Wei C, Lu Z (2020). BioConceptVec: Creating and evaluating literature-based biomedical concept embeddings on a large scale. PLoS Comput Biol.

[4] Lee J, Yoon W, Kim S, Kim D, Kim S, So CH, Kang J (2020). BioBERT: a pre-trained biomedical language representation model for biomedical text mining. Bioinformatics.

[5] Wajsbürt P, Sarfati A, Tannier X (2021). Medical concept normalization in French using multilingual terminologies and contextual embeddings. J Biomed Inform.

[6] Grabar N, Grouin C, Section Editors for the IMIA Yearbook Section on Natural Language Processing (2019). A Year of Papers Using Biomedical Texts: Findings from the Section on Natural Language Processing of the IMIA Yearbook. Yearb Med Inform.

[7] Pakhomov S, McInnes B, Adam T, Liu Y, Pedersen T, Melton GB (2010). Semantic Similarity and Relatedness between Clinical Terms: An Experimental Study. AMIA Annu Symp Proc.

[8] Pakhomov SVS, Pedersen T, McInnes B, Melton GB, Ruggieri A, Chute CG (2011). Towards a framework for developing semantic relatedness reference standards. J Biomed Inform.

[9] Mathôt S, Schreij D, Theeuwes J (2012). OpenSesame: an open-source, graphical experiment builder for the social sciences. Behav Res Methods.

[10] Pedersen T, Pakhomov SVS, Patwardhan S, Chute CG (2007). Measures of semantic similarity and relatedness in the biomedical domain. J Biomed Inform.

[11] Mikolov T, Sutskever I, Chen K, Corrado G, Dean J (2013). Distributed Representations of Words and Phrases and their Compositionality. Cornell University.

[12] Bojanowski P, Grave E, Joulin A, Mikolov T (2017). Enriching Word Vectors with Subword Information. Cornell University.

[13] Eunjeonhan project.

[14] Rehurek R, Sojka P (2010). Software Framework for Topic Modelling with Large Corpora.

[15] Shrout PE, Fleiss JL (1979). Intraclass correlations: uses in assessing rater reliability. Psychol Bull.

[16] Jiang S, Wu W, Tomita N, Ganoe C, Hassanpour S (2020). Multi-Ontology Refined Embeddings (MORE): A hybrid multi-ontology and corpus-based semantic representation model for biomedical concepts. J Biomed Inform.

[17] Pakhomov SVS, Finley G, McEwan R, Wang Y, Melton GB (2016). Corpus domain effects on distributional semantic modeling of medical terms. Bioinformatics.

[18] Bill RW, Liu Y, McInnes BT, Melton GB, Pedersen T, Pakhomov S (2012). Evaluating semantic relatedness and similarity measures with Standardized MedDRA Queries. AMIA Annu Symp Proc.

[19] Resnik P (1995). Using information content to evaluate semantic similarity in a taxonomy. https://arxiv.org/abs/cmp-lg/9511007.

[20] Lin D (1998). An Information-Theoretic Definition of Similarity. http://www.mathcs.emory.edu/~choi/courses/reading/lin-98a.pdf.

[21] Dynomant E, Lelong R, Dahamna B, Massonnaud C, Kerdelhué G, Grosjean J, Canu S, Darmoni SJ (2019). Word Embedding for the French Natural Language in Health Care: Comparative Study. JMIR Med Inform.

[22] Soares F, Villegas M, Gonzalez-Agirre A, Krallinger M, Armengol-Estapé J (2019). Medical word embeddings for Spanish: development and evaluation.

[23] Huang EW, Wang S, Lee DJ, Zhang R, Liu B, Zhou X, Zhai C (2017). Framing Electronic Medical Records as Polylingual Documents in Query Expansion. AMIA Annu Symp Proc.

[24] Wajsbürt P, Sarfati A, Tannier X (2021). Medical concept normalization in French using multilingual terminologies and contextual embeddings. J Biomed Inform.

[25] Zhang ZC, Zhang MY, Zhou T, Qiu YL (2020). Pre-trained language model augmented adversarial training network for Chinese clinical event detection. Math Biosci Eng.

[26] Pires T, Schlinger E, Garrette D (2019). How Multilingual is Multilingual BERT?.

[27] Conneau A, Khandelwal K, Goyal N, Chaudhary V, Wenzek G, Guzmán F, Grave E, Ott M, Zettlemoyer L, Stoyanov V (2020). Unsupervised Cross-lingual Representation Learning at Scale.

[28] Bender EM (2009). Linguistically Naïve != Language Independent: Why NLP Needs Linguistic Typology. https://www.aclweb.org/anthology/W09-0106.pdf.

[29] Schnabel T, Labutov I, Mimno D, Joachims T (2015). Evaluation methods for unsupervised word embeddings.

